# In vivo study of *Plasmodium falciparum* chloroquine susceptibility in three departments of Haiti

**DOI:** 10.1186/s12936-017-1961-2

**Published:** 2017-08-04

**Authors:** Christian P. Raccurt, Philippe Brasseur, Micheline Cicéron, Dana M. Parke, Marcus J. Zervos, Jacques Boncy

**Affiliations:** 1 Laboratoire National de Santé Publique, Rue Chardonnier #2 and Delmas 33, Port-au-Prince, Haiti; 2 Institut de Recherches pour le Développement, Campus international UCAD-IRD de Hann, Route des Pères Mariste, Dakar, Senegal; 3 The Global Health Initiative, Henry Ford Health System, 440 Burroughs Street, Suite 229, Detroit, MI 48202 USA

**Keywords:** Malaria, *Plasmodium falciparum*, Chloroquine susceptibility, Haiti

## Abstract

**Background:**

Malaria is considered a public health priority in Haiti, with a goal to eliminate by year 2020. Chloroquine is the first-line treatment recommended by the Ministry of Public Health and Population. In order to verify the suitability of chloroquine for uncomplicated malaria treatment, an in vivo study of susceptibility of *Plasmodium falciparum* to chloroquine was conducted from January 2013 to March 2015 in six localities in the south of Haiti.

**Results:**

Sixty-one patients who presented with confirmed *P. falciparum* malaria were included in the study and followed until day 28 after having taken 25 mg/kg of chloroquine orally over 3 days. The sample included 28 children under the age of 10, 9 adolescents aged 10–19 years, and 24 adults aged 20 years and over. Among them, 30 were monitored on day 3 (49%) and 33 on day 28 (59%). Clinical and parasitological monitoring was carried out on day 7 on 28 subjects, on day 14 on 13 subjects and on day 21 on 18 subjects. Residual parasitaemia with presence of trophozoites was found in 7 of 30 subjects on day 3 (23%), and in 6 of 28 subjects on day 7 (21%) who had a temperature less than 37.5 °C. These patients can be considered as late parasitological failures. All monitoring performed on day 28 was negative. Gametocytes were found in 3 patients (9%) despite the use of primaquine. The continuing low parasitaemia on day 3 and 7 in more than one fifth of cases raises the question of the efficacy of chloroquine in southern Haiti.

**Conclusions:**

Results suggest a decrease of chloroquine susceptibility for treatment of *P. falciparum* malaria cases in southern Haiti. Consequently, there is a need to strengthen malaria treatment surveillance and to study the effectiveness of chloroquine in Haiti by monitoring patients after treatment.

## Background

Malaria is considered a public health priority in Haiti [1–4], even though the transmission rate is low [5]. In Haiti, infections are believed to be entirely due to *Plasmodium falciparum,* and strains are believed to be susceptible to chloroquine, which is still often used in therapy, and remains the first-line treatment recommended by the Ministère de la Santé Publique et de la Population (the Ministry of Public Health and Population). Surveillance information of patients treated for malaria in Haiti is very limited. Recent studies have found *P. falciparum* isolates carrying chloroquine resistant genes [6, 7]. Additionally, therapeutic failures of oral chloroquine treatment have been reported [8]. In order to verify the suitability of chloroquine for uncomplicated malaria treatment, a study of in vivo susceptibility of *P. falciparum* was conducted from January 2013 to March 2015 in three departments in Haiti (Ouest, Sud-Est, Grande Anse).

## Methods

### Study sites

**Fig. 1:**
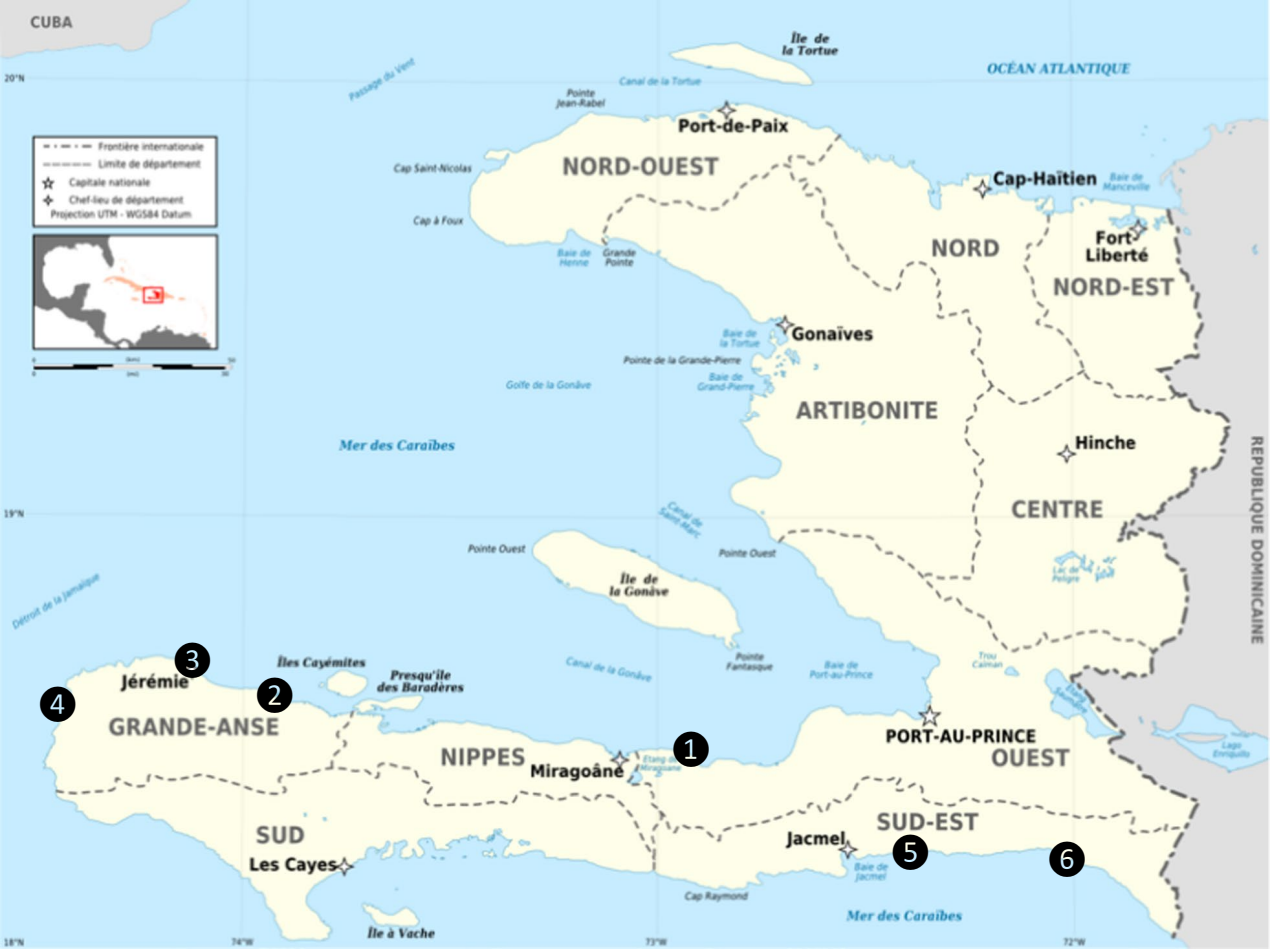
Location of sites where subjects were included in the study of chloroquine susceptibility of *Plasmodium falciparum* in three departments of the south of Haiti: *1* Petit-Goâve, *2* Corail, *3* Jérémie, *4* Bariadelle, *5* Cayes-Jacmel, *6* Anse-à-Bœuf

The study was carried out in six coastal sites in three geographic departments in Haiti (Fig. 1). In these six sites, patients were recruited from two hospitals: Hôpital Notre-Dame in Petit-Goâve (Ouest Department) and Hôpital Saint-Antoine in Jérémie (Grande Anse Department); from three community health centres: Cayes Jacmel (Sud-Est Department), Corail and Bariadelle (Grande Anse Department); and from a village known as a place of high transmission, remote from any health structure: Anse-à-Bœuf (Sud-Est Department).

### Subjects

Study participation included febrile patients and villagers with an axillary temperature greater or equal to 37.5 °C at baseline, and those who had a fever with chills within the prior 24 h. Inclusion criteria were: *P. falciparum* mono-infection with a parasitaemia ≥to 1000 asexual forms per microlitre of blood (evaluation for mono-infection was by direct microscopy), axillary temperature ≥37.5 °C on the day of the survey or fever and chills within the prior 24 h, no signs of severe or complicated malaria, and no anti-malarial treatment within the previous 2 weeks. Study objectives were explained in Haitian Creole and patients were included after having given their written consent, or that of a parent or legal guardian for minors.

A rapid diagnostic test (RDT) for *P. falciparum* and non-*P. falciparum* malaria (First Response^®^-CareStartTM Malaria Rapid Test) was performed. Questioning and a clinical examination looking for signs of malaria were conducted by the same physician (CPR) among the positive patients included in the study. For each subject, a capillary blood sample was taken from the fingertip to make a thick and thin blood smear. Research, identification and counting of sexual and asexual forms of *P. falciparum* were conducted at 1000× magnification. Subjects with less than 1000 asexual forms per microlitre were excluded from the study. Blood was collected on Whatman 903^®^ filter paper for genotyping of the parasite strain (results not yet published). Patients were treated with chloroquine and primaquine according to dosage recommended by the Ministry of Public Health and Population, namely, 600 mg of chloroquine and 60 mg of primaquine at baseline (D0) and 300 mg of chloroquine on day 1 and 2 for adults. Children received 10 mg/kg at baseline and 5 mg/kg on day 1 and 2. After administration of treatment, patients were kept under observation for 30 min to verify absence of vomiting. In case of vomiting before 30 min passed, a new dose was administered.

Whenever possible, subjects were monitored on day 1, 2, 3, 7, 14, 21 and 28, axillary temperature was monitored, a clinical exam and a thick smear were performed. The criteria for assessing treatment efficacy were those defined by the World Health Organization (WHO) [9] to distinguish adequate clinical and parasitological response including early failure, late clinical failure, and late parasitological failure.

### Laboratory procedures

Thick and thin blood smears were stained using the Giemsa method and examined under a microscope. The count of asexual forms of *P. falciparum* was performed compared to 200 leukocytes and evaluated by microlitre of blood while considering a number of 8000 leukocytes per microlitre of blood.

## Results

A total of 71 volunteers presenting with malaria due to *P. falciparum* detected by a fever greater than 37.5 °C and/or a positive RDT confirmed by microscopy of the parasite on the thick smear were enrolled. Only 61 patients met the inclusion criteria, nine having too low of parasitaemia levels (between 261 and 933 trophozoites/µL), and one subject was lost after day 1. The 61 retained subjects came from six sites across three geographic regions (departments) in the south of Haiti (Table 1). 33 subjects were male and 28 were female. There were 28 children less than 10 years old, the youngest being 18 months old; 9 adolescents aged 10–19 years; and 24 adults over 20 years of age (Table 2).

**Table 1:** Sites in which subjects were recruited and the number of subjects reviewed at each monitoring

Department	City	Date	Number of subjects enrolled	Number of subjects examined at each monitoring						
			Day 0	Day 1	Day 2	Day 3	Day 7	Day 14	Day 21	Day 28
Ouest	Petit-Goâve	January 2013	1	NA	NA	1	NA	NA	NA	1
Grande Anse	Corail	January 2013	11	NA	NA	6	NA	NA	NA	6
Grande Anse	Jérémie	February 2013	2	NA	NA	0	NA	NA	NA	1
Sud Est	Cayes Jacmel	March 2013	3	NA	NA	0	NA	NA	NA	2
Grande Anse	Corail	May 2013	2	NA	NA	1	NA	NA	NA	0
Sud Est	Anse-à-Bœuf	January 2014	9	5	5	3	6	7	7	8
Sud Est	Anse-à-Bœuf	February 2014	5	NA	NA	0	4	3	NA	NA
Sud Est	Anse-à-Bœuf	June 2014	4	4	4	4	4	3	3	3
Grande Anse	Bariadelle	February 2015	24	21	17	15	14	NA	8	12
Totals			61	30	26	30	28	13	18	33
				49%	70%	49%	67%	72%	49%	59%

*NA* not applicable

**Table 2:** Distribution of subjects by sex and age

Age (years)	Male	Female
≥50	3	3
20–49	9	9
10–19	6	3
1–9	15	13
Total (range 1–75)	33	28

The parasitic density of asexual forms from the 61 monitored subjects varied from 1000 to 277,227 trophozoites/µL (mean: 48,811.5; median: 14,686). The distribution of patients according to age and parasitic density is given in Table 3. The three patients with the highest levels of parasitaemia were, respectively, 4 years old (201,538 trophozoites/µL), 5 years old (277,227 trophozoites/µL) and 7 years old (262,223 trophozoites/µL).

**Table 3:** Distribution of subjects by age and parasitic density of trophozoites of *Plasmodium falciparum* per microlitre

Age (years)	Parasitic density of trophozoites/microliters of blood			
	1000–9999	10,000–49,999	50,000–149,000	150,000–300,000
≥50	1	3	2	0
20–49	9	4	4	2
10–19	2	1	3	2
1–9	13	7	4	4
1–75	25 (41%)	15 (25%)	13 (21%)	8 (13%)

Of the 61 patients included, 30 were monitored on day 3 (49% of the sample). Seven (23.3%) still showed *P. falciparum* trophozoites in the thick smear, however, in a smaller quantity than on day 0 and without clinical signs (temperature <37.5 °C). Six out of 28 still had *P. falciparum* throphozoites on day 7, but with an axillary temperature <37.5 °C.

By monitoring at a distance, 13 patients were reviewed at day 14, 18 patients at day 21, and 33 patients at day 28 at the end of the study. Two 2-year-old children showed persistence of trophozoites in the thick smear until day 21, but with an axillary temperature <37.5 °C. At day 28, all 33 controlled subjects had a negative thick smear without trophozoites and an axillary temperature <37.5 °C. Only 3 of them were still gametocyte carriers. During clinical follow-up, we observed the appearance on day 28 of a level 1 spleen according to the Hackett classification in a 6-year-old girl and a 72-year-old woman.

## Discussion

The results of this study showed a decrease of chloroquine susceptibility for treatment of *P. falciparum* malaria cases in southern Haiti. Six patients failed therapy. As chloroquine is inexpensive and commonly used in Haiti this study has important implications. As stated by the World Health Organization [10], high parasitaemia is a risk factor for death from malaria. Hyperparasitaemia >250,000 parasites/µL is an additional criteria for severe malaria in non-immune individuals. The relation between parasitaemia and prognosis varies according to the level of malaria transmission. As Haiti is a low-transmission area [1, 5], it seems probable that mortality from acute falciparum malaria increases with parasite densities over 100,000/µL.

Close monitoring of patients during treatment resulted in a rapid decrease of parasitic density in four out of five cases. Two 2-year-old children in Anse-à-Bœuf retained trophozoite parasitaemia on day 14 and 21, but not on day 28 when only gametocytes were found. Throughout the entire follow-up period, their axillary temperature was <37.5 °C. For one patient, parasitaemia decreased dramatically, going from 3579 trophozoites/µL at baseline to 204/µL at day 14–44/µL at day 21. For the other, there were 1280 trophozoites at baseline and parasitaemia remained at this same level on day 7, 14, and 21. These two cases can thus be considered as two late parasitological failures.

Among all patients, the majority or most of the clinical symptoms noted on day 0 disappeared as early as the day 2 or 3 monitoring, except for the pallor of the mucous membranes, attesting to the frequent anemia among children in these villages. The fever decline was rapid: while nearly half of the patients had an axillary temperature between 37.5 °C and 40.8 °C at baseline, only two of 31 patients had, respectively, an axillary temperature of 37.9 °C and 38.1 °C on day 1, only one out of 25 on day 2 (37.5 °C), and one out of 30 on day 3 (38.1 °C). Afterward, all patients that were monitored weekly until day 28 had an axillary temperature of less than 37.5 °C.

Through questioning and examination, the improvement of clinical status was observed among all patients during monitoring, indicating that chloroquine remains effective in Haiti. This study shows that the majority of *P. falciparum* isolates remain susceptible, in vivo, to chloroquine in Haiti, as was observed some 30 years ago [11, 12] and until recently in Léogane among 49 patients [13].

However, in a recent study [8], the persistence of parasitaemia among 7 out of 60 asymptomatic patients monitored until day 28 and 42 was observed in the Ouest department, and interpreted as late parasitological failures. These recent findings are similar to the results of this study. They likely indicate an onset of decreased susceptibility of this drug in the treatment of malaria in Haiti. These in vivo results should be compared with recent detection of *P. falciparum* haplotypes carrying chloroquine resistant genes in Artibonite [6].

Two major difficulties were encountered during this study. The first was the recruitment of cases that met the pre-established inclusion criteria. It is currently difficult to recruit malaria cases in Haiti, except in a few specific areas such as Anse-à-Bœuf in the Sud-Est department (N°6 on the map in Fig. 1), and Bariadelle in the Grande Anse department (N°4). This is why these two sites were prioritized to conduct this study, despite their distance from Port-au-Prince, and the difficulty of accessing Anse-à-Bœuf. As for the majority of febrile subjects examined during visits to the health centres, they tested negative by RDT. This confirms that malaria is not the leading cause of febrile illness in Haiti. It is, therefore, essential to achieve precise diagnosis of fevers using the increasingly available rapid tests, to avoid unnecessarily treating all febrile subjects with anti-malarials.

The second limitation concerns the routine monitoring of included subjects. Despite their consent to participate in the study, some subjects were lost and did not return to their follow-up appointments, which resulted in variation of the days where follow-up monitoring was performed. This loss of patients was experienced less in the fishing villages of Anse-à-Bœuf and Bariadelle, where it was easier to find the subjects in their home, in case they missed monitoring appointments. However, despite this limitation, the study still indicates the decrease of chloroquine susceptibility by *P. falciparum* parasites.

## Conclusions

The implications of this study highlight the need to strengthen surveillance of decreasing in vivo susceptibility of *P. falciparum* throughout the country in order to identify the regions affected by these failures in chloroquine treatment. This information is critical in determining appropriate therapeutic and control measures in Haiti.

## Abbreviations

RDT: rapid diagnostic test
WHO: World Health Organization
